# Inflammatory Skin Disorders: Monocyte-Derived Cells Take Center Stage

**DOI:** 10.3389/fimmu.2021.691806

**Published:** 2021-07-14

**Authors:** Heena Mehta, Julianty Angsana, Robert Bissonnette, Ernesto J. Muñoz-Elías, Marika Sarfati

**Affiliations:** ^1^ Immunoregulation Laboratory, Centre de Recherche du Centre Hospitalier de l’Université de Montréal (CRCHUM), Montréal, QC, Canada; ^2^ Immunology, Janssen R&D LLC, San Diego, CA, United States; ^3^ Innovaderm Research, Montréal, QC, Canada

**Keywords:** monocytes, macrophages, scRNA-seq, flow cytometry (FCM), skin - immunology

## Introduction

Current biologics targeting pro-inflammatory cytokines in psoriasis and atopic dermatitis (AD) show excellent clinical efficacy but are not curative, underscoring the need to study the skin immune and non-immune landscape. A recent article published in *Science* employed single-cell RNA sequencing (scRNA-seq) to generate an atlas of healthy human skin during early prenatal life and adulthood, and further dissect changes occurring in inflammatory skin diseases (1). Developing skin is enriched in innate lymphoid cells and macrophages (Mϕ) while healthy adult skin is predominantly populated with T cells, Langerhans cells (LCs), and dendritic cells (DCs). Specifically, adult skin Mϕ were subdivided into Mac1 and Mac2 based on distinct molecular signatures. In AD and psoriasis, the percentage of Mac2 is increased resulting in an altered Mac1/Mac2 ratio in lesions. Fibroblasts predominate in fetal non-immune cell compartment while keratinocytes, melanocytes, and endothelial cells represent the major cell types in healthy adult skin. Among the three vascular endothelial (VE) cell subsets identified, only the proportion of VE3 is elevated in adult inflamed skin. Interestingly, Mac2 and VE3 in inflamed skin share a common molecular signature with Mϕ and VE in fetal skin. Overall, the authors conclude that skin developmental programs are recalled during inflammatory skin diseases and proposed Mϕ as potential targets of treatment. The concept of re-emergence of fetal skin program was recently reported for fetal Tregs that express an effector memory phenotype and closely align with adult Tregs in healthy skin (2). The authors further highlight the advantage of this molecular approach that identified 14 mononuclear phagocyte (MNP) cell states, and state that flow cytometry (FCM) has limitations to uncover diversity of rare cells. However, high dimensional single cell FCM is a complementary approach that has been proven suitable to reveal heterogeneity of skin MNP cell states in blood and skin (3, 4).

## Need for Consensus in Mononuclear Phagocyte Nomenclature

**Figure 1:**
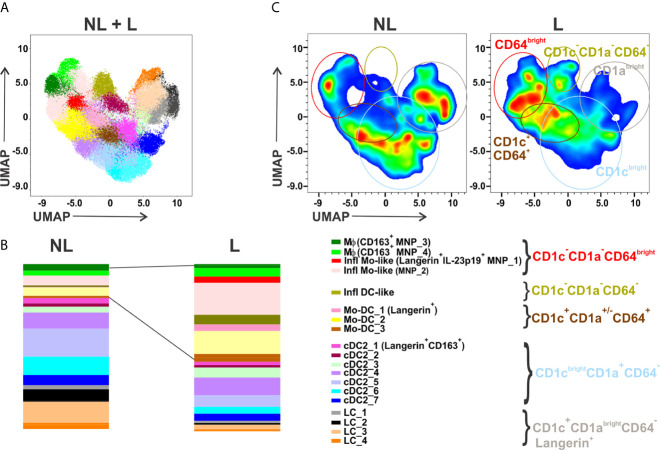
This figure is another representation of flow cytometry data published in Mehta et al. *(Journal of Investigative Dermatology 2021)*. MNPs gated as CD45^+^CD11c^+^HLA-DR^+^ cells from matched NL and L skin of patients with psoriasis (n = 20) were concatenated for unsupervised analysis. Parameters included in analysis were CD11c, HLA-DR, CD14, CD64, CD163, CD1c, CD1a, CD207, FcεRIα, IL-23p19 and TNFα. Dimensionality reduction was performed by UMAP and clustering by FlowSOM. **(A)** FlowSOM applied on concatenated CD45^+^CD11c^+^HLA-DR^+^ MNPs identified 19 cell states which were overlaid on combined NL and L UMAP plot. **(B)** Relative distribution of MNP cell cluster frequencies identified in **(A)** in NL *versus* matched L psoriasis skin. **(C)** UMAP plots showing the increased and decreased areas identified in **(B)** in L compared to matched NL psoriatic skin. UMAP, Uniform Manifold Approximation and Projection; NL, non-lesional; L, lesional; MNPs, mononuclear phagocytes; SOM, self-organizing map. Note that antigen markers for detection of cDC1 and plasmacytoid DCs were not included in this study.

Identification of a phenotypic or transcriptomic signature to precisely define MNP subsets in inflamed tissues has resulted in confounding nomenclature. Monocytes recently recruited to inflamed skin have been referred to as monocyte-derived dendritic cells (Mo-DC), inflammatory DC, inflammatory monocyte-like (Infl Mo-like) cells, inflammatory Mϕ (Inf mac), or monocyte-derived Mϕ (Monomac) highlighting a need to better define their molecular identities and function (5). Also, circulating conventional DC2 (cDC2) subsets reflect a continuum of activation states which remain unclear in inflamed skin (3). Our recently reported FCM analysis, that utilized nine surface and two cytokine markers with the aim to identify IL-23 or TNFα- expressing MNPs (4), revealed MNP heterogeneity in psoriatic non-lesional (NL) and lesional (L) skin. Another representation of these previously reported data using a different clustering algorithm, FlowSOM, discerned 19 MNP clusters: two Mϕ, two Infl Mo-like cells, one inflammatory DC-like (Infl DC-like), three Mo-DC, seven cDC2 and four LC subsets (**Figure 1**). As depicted in **Figures 1B, C**, frequencies of Infl Mo-like MNPs which included the IL-23-expressing subset, Infl DC-like and Mo-DCs were augmented while cDC2 and LC subsets were decreased in L skin.

The altered Mac1/Mac2 ratio identified by scRNA-seq in skin lesions in Reynolds et al. might corroborate with decreased CD14^bright^CD64^bright^CD163^+^ MNP_3 and increased CD14^dim^CD64^bright^CD163^+^ MNP_4 Mϕ clusters detected by FCM in psoriatic L skin (**Figure 1B**). Interestingly, Xue et al. identified three CD163^+^ Mϕ cell states in healthy skin by scRNA-seq and immunofluorescence on tissue sections, resident-like MARCO^+^ Mϕ, inflammatory-like CCR1^+^ Mϕ and TREM2^high^ Mϕ (6). The relationship between Mac1 (*CD14*, *MARCO*, *CD163*), Mac2 (*F13A1*, *CD163*), the three CD163^+^ Mϕ reported by Xue et al., and the two MNP_3 and MNP_4 CD163^+^ Mϕ clusters warrants further investigation. Notably, *F13A1* also belongs to the signature of resident-like MARCO^+^ Mϕ (6), and the Mϕ cluster (*CD163*, *CD14*) in biopsy but not suction blister sample in AD (7). These differences underline the need to align sample collection, cell isolation and data curation methodologies to arrive at a consensus for nomenclature.

## Inflammatory Monocyte-Like Cells Are the Major MNP Subset in Inflamed Skin

It must be emphasized that CD14^bright^CD64^bright^CD163^-^ Infl Mo-like cells (MNP_1 and MNP_2) represent the major clusters increased in L skin that were significantly reduced in healed skin during treatment with guselkumab (anti-IL-23p19) or secukinumab (anti-IL-17A) (4). In contrast, the two Mϕ clusters, MNP_3 and MNP_4, like Mac1 and Mac 2 (1), represent relatively minor MNP subpopulations in L skin (4) (**Figure 1B**). Reynolds et al., in Figure S5, further show that CD1a^lo^Ki-67^+^ cells expressing Langerin (CD207) infiltrate inflamed skin. In fact, these cells might comprise CD207^+^CD1a^-^ Infl Mo-like (MNP_1) cells as well as CD207^+^CD1a^-^ Mo-DC_1 that are distinct from CD1a^bright^CD207^+^ LCs (**Figure 1**). Interestingly, proportion of LCs, drastically reduced in L skin, was restored during guselkumab or secukinumab treatment (4) supporting their ability to self-renew (8). Alternately, LCs could originate from recruited monocytes in healing tissue (9). Noteworthy, the data from Figure S5 in Reynolds et al. suggesting that a cell state molecularly defined as Inf mac, but not Mac2 or Monomac, is a major subset that significantly increased in L psoriasis skin, are not discussed by the authors. We propose that dermal Inf mac which appear to express *IL23A* but not *CD14* in adult healthy skin, and Monomacs displaying a monocyte-like molecular signature (*CD14, S100A8, S100A9*) comprise a mixture of Infl Mo-like MNP_1 and MNP_2 clusters identified by FCM. These dermal MNPs are likely not steady-state tissue-resident Mϕ but derived from monocytes recruited in skin during inflammation and contribute to disease pathogenesis (8). Whether MNP_1 and MNP_2 further differentiate into Mϕ remains to be elucidated. Finally, a cell interaction model described in Reynolds et al., predicted that Mac2-derived CXCL8 interacting with ACKR1^+^VE3 cells is the key pathway contributing to skin inflammation. However, Inf mac, Monomac and MoDC1 also express *CXCL8* (Table S1 in Reynolds et al.) and can interact with VE3 as well highlighting that the contribution of other MNP subsets cannot be ignored, and furthermore, warrants functional validation.

## Concluding Remarks and Future Directions

Unsupervised FCM analyses strongly support and complement scRNA-seq data for uncovering immune cell heterogeneity and complexity in tissue (skin, lung, gut, liver, heart, brain). Cytometry offers the advantage to appreciate cellular activation status based on expression levels of markers in tissue as well as polyfunctional status of cells in terms of secretion of cytokine, chemokine and effector molecules. This was recently demonstrated in different infectious disease models to define specific T helper profiles (10). scRNA-seq techniques can define a complete molecular identity card for cells that can be utilized by bioinformatics to predict cellular function (pathway analysis), interactions and connectivity (progression analysis) which would need to be functionally validated. Techniques that combine the strengths of both methodologies such as CITE-seq (Cellular Indexing of Transcriptomes and Epitopes by Sequencing) may help to connect cellular phenotypes with molecular signatures and function in inflamed tissue.

Longitudinal study of tissue samples in a homogenous group of patients, as opposed to snapshot analysis in different cohorts, helps to identify potential pathogenic and regulatory cells, as well as key targets of treatments. Indeed, following the same psoriasis lesion before and during treatment at different time points strongly suggests that the major Infl Mo-like cells were the target of therapeutic cytokine blockade, underscoring their key role in disease pathogenesis (4).

Finally, further studies on human monocyte cell fate in inflamed and non-inflamed tissue warrant consideration. A consensus in nomenclature that may help to distinguish monocytes in transition to macrophages versus tissue-resident macrophages, distinct from monocytes differentiating into dendritic cells, should encompass the nature and nurture, plasticity, function of these cells and not be simply based on gene expression. This would greatly facilitate advancement/dissemination of scientific knowledge by avoiding calling subsets by different names when reporting a subset with similar phenotypic, molecular and functional signature.

Overall, uncovering the cues for monocyte-derived cell plasticity toward MNP subsets endowed with regulatory, repair or anti-inflammatory function that restore skin homeostasis might open avenues to develop novel therapeutic strategies for these two common inflammatory skin disorders.

## Author Contributions

MS and HM wrote the first draft of the manuscript. All authors contributed to the article and approved the submitted version.

## Conflict of Interest

RB is an employee and shareholder of Innovaderm Research. JA and EJME are/were employees of Janssen Research & Development, a wholly owned subsidiary of Johnson & Johnson. JA and EJME may own stocks in Johnson & Johnson.

The remaining authors declare that the research was conducted in the absence of any commercial or financial relationships that could be construed as a potential conflict of interest. 
